# Factors Associated with Relapses in Alcohol and Substance Use Disorder

**DOI:** 10.5152/eurasianjmed.2023.23335

**Published:** 2023-12-01

**Authors:** Ahmet Bülent Yazıcı, Muhammed Raşit Bardakçı

**Affiliations:** 1 Department of Psychiatry, Sakarya University Faculty of Medicine, Sakarya, Turkey; 2 Sakarya Training and Research Hospital, Sakarya, Turkey

**Keywords:** Addiction, alcohol, relapse, substance

## Abstract

Alcohol and substance use disorder (ASUD) is a chronic condition featuring relapses and remissions. Due to their multifactorial nature, the causes of relapses in ASUD are not fully understood and it is important to update the information. Therefore, we aimed to provide an update on research examining factors associated with relapses, organized into sections. Factors such as early age of onset, dysfunction in the brain reward system, poor physical health, sleep disturbance, comorbid psychiatric disorders, severity of ASUD, craving, low self-efficacy, negative life events, and low socioeconomic status have been consistently shown to increase the relapse rate of ASUD. Conversely, factors such as positive family functioning, strong social support, treatment motivation and regular medication appear to decrease relapse rates. Studies on gender and genetic factors have yielded mixed results and no consistent relationship with relapses has been found. While pharmaceutical agents such as methadone, buprenorphine and naltrexone are effective in preventing relapse in opioid and alcohol use disorders; there are no agents proven to be effective in other substance use disorders. Psychotherapeutic approaches, including motivational interviewing, cognitive behavioral therapy, 12-step programs and contingency management have been demonstrated to be effective. The treatment of addiction to substances is intricate and necessitates biopsychosocial interventions. However, despite all treatments, high relapse rates indicate the necessity for social support and rehabilitation services. In the light of the data obtained, the factors affecting relapses in patients with ASUD can be determined and appropriate interventions and therapeutic approaches can be used to prevent relapses.

## Introduction

Alcohol and substance use disorder (ASUD) is a significant problem that affects individuals, families, and communities and is associated with many psychological and medical issues.^1^ Formerly, the term addiction is used and nowadays still this term is a broad concept and can be extended from alcohol and substance addiction to behavioral addictions and food addiction and more, which is not included in the classification systems.^2-4^ Here we will prefer to use ASUD where it is available.

Alcohol and substance use disorder is a persistent illness characterized by remissions and relapses.^5^ Relapse rates are found to be similar across various substance addictions, with many people returning to substance use within the first 3 months following treatment and relapse rates of around 70% at 1 year following treatment.^6,7^ One study on relapse in patients with ASUD found that only 39% of patients were able to remain in remission during the 1-year follow-up period,^8^ while another study found that 50%-60% of patients relapsed within a few months after detoxification. In a study carried out in Turkey, relapse rates were found to be 61.8% after a 1-year follow-up, with another study indicating a 60.8% relapse rate after a 6-month follow-up.^9^ Notably that relapse rates in AMKB are consistently high across various populations and time periods.

Prevention of relapses requires knowledge of the factors that trigger them and maintaining the remission process. Relapse rates are high in addiction, especially in the early stages, and current treatments often fail to provide sufficient benefit in the course of addiction.^10^ Therefore, determining the factors associated with relapses is of great importance. In evaluating patients in terms of relapse risk, it is necessary to pay attention to personal, situational, and physiological factors and their interactions, and to consider patients and relapse in a multidisciplinary manner.^11^ The patients’ biological, psychological, and social characteristics have been shown to impact their prognosis. It was found that a stable marriage, high socioeconomic status, and older age decreased relapse, whereas neuropsychological impairment, high addiction severity, and the presence of comorbid psychiatric symptoms increased relapse.^10^ Additionally, a history of addiction in the family, starting drug use at a young age, impulsive and risk-taking personality traits, the severity of the addiction, the presence of co-occurring psychiatric disorders, taking additional medication, difficulties in interpersonal relationships, and poor problem-solving skills all contribute to an increased risk of relapse.^12^

In the literature, there are review articles that examine the factors associated with relapses in alcohol use disorder (AUD) or substance use disorder (SUD) separately. Our study examined these factors in both AUD and SUD in the light of current data. Detailed identification and recognition of the remission and relapse periods of individuals with alcohol and substance dependence and the factors that cause these periods seem to be of great importance for the development of individualized treatments, early medical and psychological interventions, and prevention of relapses. The aim of this review article aims to summarize the factors associated with relapses in ASUDs and to present current developments in the field.

## Clinical and Research Consequences

Based on the data in the literature and previous similar studies, the factors affecting relapses were analyzed under the headings of Sociodemographic Factors, Brain Imaging and Genetic Factors, Clinical Factors, and Social Factors.^10,13^

### Sociodemographic Factors

#### Age

Age-related variables have been associated with relapses in 2 aspects. The first is the age of onset of alcohol or substance use and the other is the chronological age at the time of the study. In a study conducted in Singapore with 6616 people from different ethnic groups, older age at onset was associated with a higher likelihood of remission in 15 people with alcohol use disorder.^14^ In another study, lower age at onset was associated with higher relapse rates in 2820 adults with major depressive disorder and AUD comorbidity.^15^ In a review, age was found to have a statistically significant effect on recurrence in 31 studies, while age had no statistically significant effect on recurrence in 15 studies.^13^ The association between early onset of alcohol use and the risk of dependence and relapses has been shown in several studies. In a study of 4778 people with alcohol use disorder (AUD), early initiation of alcohol use was associated with a higher risk of relapse, which appears to be associated with a higher risk of developing dependence at an earlier age.^16^ The findings of this study were supported by a small-scale study that reported that the age of onset of addiction, but not the age of use, was associated with the duration of development.^17^

Although there are conflicting results when the results of the studies are evaluated in general, older age of onset can be associated with higher remission and earlier age of onset with higher relapse. However, studies investigating the relationship between age of onset and relapses have been conducted in very different populations and in patients with different psychiatric and medical comorbidities.^13^

In a study on SUDs, early age of onset was associated with polysubstance use but not with relapses.^18^ In a study of 4175 opioid addicts on methadone maintenance treatment, low age at first substance use was associated with relapses.^19^ Epidemiologic studies show that a younger age at first substance use is associated with an increased risk of SUDs and an increased rate of relapse.^20^ These results suggest that the effects of age at first intervention on relapses are similar in AUD and SUD.

#### Gender

Although alcohol use and dependence predominate in men, gender has not consistently predicted relapse in alcohol use disorder studies.^21,22^ While some studies found that female gender was significantly associated with better prognosis, more studies have not found a statistically significant relationship between gender and recurrence rates.^13^ Women in AUD were found to be more likely than men to relapse following a stressful event or a drug-related cue, although the impact on relapse rates is unclear.^23^ Additionally, a history of trauma and current trauma-related symptoms are significantly associated with relapse in women in contrast to men.^24^

Although studies showing that relapse rates are lower in women with SUDs, data on gender-specific determinants of relapse risk are limited and further studies are needed.^25^ In 2 studies, women showed lower relapse rates than men in buprenorphine-treated patients.^26^ However, it does not support the concept of gender differences for cravings and relapses for psychostimulants in general or opioids in particular.^27^ Similar to AUD, women with cocaine use disorder are more likely to attribute relapse to negative emotional states and interpersonal conflict than men.^26^

### Brain Imaging and Genetic Factors

#### Brain Imaging Studies

Brain-based predictors of relapses measured by neuroimaging in alcohol dependence and substance addictions have been examined in many studies, and the results were found to be consistent with neurobiological models of addiction. Studies have found that dysfunction in the brain reward system, nucleus accumbens, executive control network, insula, and ventral pallidum are associated with significantly higher relapse rates.^13^ In addition, it has been observed that limbic system and cerebellum hypermetabolism and frontal hypometabolism are associated with relapse in alcohol dependence.^28^ Weakened functional connections in corticolimbic and corticostriatal circuits in substance abuse have also been associated with a higher risk of relapse.^29^ Brain-derived neurotrophic factor increase in the medial prefrontal cortex may prevent relapses in AUD.^30^

One study using animal models showed that dorsal hippocampus activity is associated with relapse in SUD, and if this activity is reduced, relapses will decrease.^31^ Another study showed that ventral pallidum GABA neurons control opioid relapses in opioid addiction.^32^

#### Family History of Disease and Genetics

The association between genetic factors such as family history of disease and specific single nucleotide polymorphisms, which reflect biological and shared environment variance, and relapse in AUD has presented mixed results.^13^

Overall, although slightly more studies have found that family history of disease and genetics are associated with a higher risk of relapse, many other studies have not found an association between family history or genetic factors and the risk of relapse.^13,33^

Substance use in close relatives has been shown to increase the risk of SUD relapse, but no direct association has been found with a family history of disease and genetic factors.^34^

### Clinical Factors

#### Physical Health

The impact of health as a predictor of relapse in alcohol dependence was identified as a statistically significant predictor of relapse or remission in a metanalysis of 9 studies with a total sample of 11 541 people in a review, and poorer physical health was significantly associated with higher relapse risk in most studies.^13^ A recent study of 400 patients with opioid use disorder supports the data that poor physical health increases relapses.^35^

#### Sleep

The relationship between ASUD and sleep problems is a well-known issue and has been investigated in many aspects.^36^ Eight studies with more than 400 patients found that sleep disturbance was associated with significantly higher relapse rates, and only 1 study found that disturbance in sleep did not affect relapse.^13,37^

#### Receptors/Hormones and Biomarkers

Hormonal factors and specific biomarkers of alcohol and substance use have generally been studied less frequently and with smaller sample sizes. However, a number of studies have found that impaired hormones and elevated biomarkers are associated with relapse.^38,39^ For example, increased stress-induced craving has been associated with a blunted cortisol response, which is a predictor of shorter relapse duration in outpatients with AUD.^40^ In a study of inpatients with AUD, it was observed that the neutrophil–lymphocyte ratio was high in the relapse group.^41^

Studies are showing that plasma cortisol and dehydroepiandrosterone sulfate (DHEA-S) levels are low in cocaine withdrawal, that they can be used as biomarkers to predict relapse, and that treatment with DHEA reduces relapse.^42,43^

#### Comorbid Psychiatric Disorders

Studies show that comorbid psychiatric disorders are common in ASUD.^44^ And also similar result was found in the relationship of increased ASUD relapse in 45 studies of people with alcohol use disorders (total n = 25 076), psychiatric comorbidity, usually diagnosed as mood disorder or attention deficit hyperactivity disorder (ADHD), was significantly associated with an increased risk of relapse.^13,45^

In a study of 247 patients with ASUDs, relapse was higher in those with comorbid depression.^46^ Another study involving 911 patients found that comorbid depression increased relapse in SUDs.^47^

#### Personality Traits and Personality Disorders

The relationship between addiction and temperament and character is well established.^48,49^ In studies examining the effect of personality traits on relapse in alcohol dependence, traits such as having a neurotic personality, risk-taking tendency and low mental resilience were found to be associated with higher relapse.^50,51^ Similarly, high neuroticism and low consequences were found to be associated with high relapse in substance abusers. As a result, it can be said that high emotional instability and behavioral disinhibition are related with a relapse of heavy use of alcohol and other drugs.^52^

Antisocial and borderline personality pathologies are prominent in relation to relapse in SUD, but data are limited and randomized controlled studies with more extended follow-up periods are needed.^18^ Similarly, in a study conducted in alcohol dependence, antisocial personality disorder was found to increase the risk of relapse;^53^ some studies have not found this relationship.^13^

#### Severity of Addiction

In a study conducted with patients with ASUD, higher disease severity was found to be associated with higher relapse rates.^1^

In a study conducted with 488 patients with opioid use disorder, higher relapse rates were observed in people who injected drugs and received higher doses of buprenorphine–naloxone.^54^ In a study involving 128 patients with cocaine addiction, it was found that disease severity increased relapses.^55^

#### Craving

While most of the studies concluded that craving is an important predictor of relapses in alcohol dependence,^39,56^ a few studies did not find a significant relationship between craving and relapse.^13^ In addition, similar to AUD, there are studies showing that craving increases relapses in SUD patients.^47,57^

#### Duration of Abstinence

In alcohol dependence, studies are showing that relapses are higher in those with shorter duration of abstinence.^58,59^ Similarly, in a recent study of 488 patients with opioid use disorder, higher relapse rates were observed in those with shorter substance-free periods.^54^

#### Emotion and Emotion Regulation

Emotion regulation skill is seen as an important issue in the prevention of any kind of addiction.^60^ Emotional regulation skill in addition to the development of addiction, also related to the abstinence period of ASUD. Most studies on alcohol dependence (25 studies, n = 10 139) found that negative emotion showed a statistically significant robust effect on relapse and was associated with a greater risk of relapse.^13^ In addition, there is a study showing that difficulties in emotion regulation increase relapse.^61^ Similar results have been found in SUDs. For example, in a study conducted with patients with methamphetamine use disorder, it was shown that positive emotions during use and negative emotions when not using increased relapses.^62^

#### Self-Efficacy

Studies in patients with alcohol use disorder have shown that higher levels of self-efficacy are significantly associated with a lower risk of relapse.^13,63^

In opioid addiction, low self-efficacy was found to be significantly associated with higher relapse.^64^

Psychological resilience is an essential factor in SUD and has been found to be associated with relapses.^65,66^

#### Comorbid Substance Use Disorder

It has been shown in many studies that the presence of comorbid SUD in alcohol dependence significantly increases relapses.^13,59^ In opioid addiction, concomitant polysubstance use has been shown to increase relapses.^67^

#### Smoking

Many studies have shown that smoking increases the risk of relapse in alcohol and substance use disorders.^59,68,69^ There are few studies showing that there is no relationship between smoking and relapse.^13^

#### Stress, Negative Life Events, and Coping

Adverse life events and lower coping skills have been found to be strongly associated with the development of SUD.^70^ Similarly, adverse life events, particularly trauma and stress, have been associated with significantly higher relapse rates in alcohol dependence.^71^ The presence of adverse childhood experiences in opiate addicts under buprenorphine–naloxone treatment increases relapses despite treatment.^72^ Traumatic experiences were found to be associated with high relapse rates in a study conducted with patients with ASUD.^9^

A study conducted with 180 male patients diagnosed with SUD shows that inadequate coping skills increase relapses.^18^ There are also studies showing that inadequate coping skills increase relapses in alcohol dependence.^73^

#### Neurocognitive Deficits

Neurocognitive deficits are associated with higher relapse rates in AUD.^59^ Cognitive deficits such as impairments in learning and memory processes in heroin addicts and executive dysfunction in methamphetamine use disorder have been found to be associated with higher relapse rates.^74,75^

#### Impulsivity

In a review study on AUD, it was concluded that impulsivity increased relapses.^76^ Again, there is a study consisting of 171 patients showing that impulsivity increases relapses.^77^ In addition, in studies with smaller samples, mixed results have been obtained as impulsivity has a significant relationship with relapse or not.^78,79^

#### Motivation and Insight

In a research involving 247 patients with ASUDs, it was discovered that having a strong motivation to quit substance use can decrease the likelihood of relapses.^46^ Setting treatment goals and increasing motivation has been demonstrated to decrease relapse rates in individuals with alcohol use disorder.^80^ In conclusion, other psychological factors including motivation, insight, help-seeking, drinking goals, and negative drinking consequences were evaluated less frequently and with fewer participants in each study. However, all these factors had a significant association with relapse. Lower insight, less seeking help and motivation, as well as negative alcohol-related consequences and lower positive outcome expectations, were significantly linked to a higher risk of relapse.^13^

#### Factors Associated with Treatment

Studies on treatment that have been shown to reduce relapses in ASUDs other than the known treatment options are summarized under this heading.^13,81^

Although disulfiram seems to be more successful than naltrexone and acamprosate in preventing relapses in compliant and controlled patients, its potentially severe complications and compliance problems and methodological shortcomings of the studies overshadow this success.^82^ A meta-analysis of 122 randomized controlled trials and 1 cohort study (a total of 22 803 participants) found no difference between naltrexone and acamprosate in reducing alcohol intake.^83^ In a study involving 96 patients diagnosed with alcohol use disorder, ketamine infusion treatment administered 3 times a week was shown to reduce relapses.^84^ In another study, intestinal microbiota modification by lactobacillus and *N*-acetylcysteine–acetylsalicylic acid treatments were found to reduce relapses and heavy drinking.^85^

Methadone and buprenorphine both decrease the risk of relapse in opioid addicts. Some studies have suggested that methadone is more effective than buprenorphine for treating opioid addiction; however, the risk of abuse with methadone requires a strict schedule.^86^ Buprenorphine was found to be slightly less effective than methadone in preventing relapse. However, the fact that buprenorphine has a lower potential for abuse and its pharmacological structure, which allows patient autonomy and does not require strict monitoring, may be an advantage in its management in treatment.^87^ Finally, a recent randomized controlled trial compared the effectiveness of extended-release naltrexone and buprenorphine–naloxone combination therapy in preventing opioid relapses. Both treatments demonstrated equal safety and efficacy.^88^

Various agents have been researched to prevent cannabis relapse, but inadequate evidence limits the widespread implementation of these findings in clinical practice. Certain studies suggest that preparations containing THC can enhance treatment completion rates. However, trials involving bupropion, selective serotonin reuptake inhibitors, mood stabilizers, anticonvulsants, gabapentin, and *N*-acetylcysteine have yielded insufficient results to support their utilization in treatment.^89^

Many pharmacological agents have been tried in the treatment of methamphetamine use disorder. These include psychostimulants, antidepressants, antipsychotics, acamprosate, naltrexone, topiramate, baclofen, gabapentin, *N*-acetylcysteine, oxazepam, atomoxetine and oxytocin. However, none have shown a clear effect on relapse.^90^ A 12-week follow-up study revealed that those with moderate to severe methamphetamine use disorder responded better to a combination of extended-release injectable naltrexone and daily oral extended-release bupropion compared to placebo in terms of relapses. Nevertheless, the response rate in the drug group remained low, at just 13.6%.^91^

In studies examining the effects of mindfulness-based intervention on relapse in SUD, although there are studies showing that it may reduce relapses, no significant effect on relapse was shown in a meta-analysis of 9 RCTs of mindfulness-based relapse prevention study.^92^

A study comparing the 1-year effectiveness of 12-step treatment and cognitive therapy found that although 12-step treatment was slightly more effective in terms of substance withdrawal, both were similarly effective in many areas, such as functioning and frequency of substance use.^93^

The meta-analysis study showed that contingency management reduced substance use in the first 3 months, and motivational interviewing had a similar effect in the following 3-6 months. The reductions were most prominent for cannabis, tobacco, and stimulants but smallest for polysubstance use.^94^

In a recent study, patients with ASUD who received psychoeducation and a music intervention effectively reduced stress, increased self-efficacy, and reduced relapse compared to controls after 6 months of follow-up.^95^

### Social Factors

Many studies on alcohol abusers have found that social factors and the quality of social support may be associated with reduced relapse risk and that having a favorable social context and functioning (e.g., employment, higher socioeconomic status, education) is associated with reduced relapse risk.^96,97^ In a study with 926 alcohol addicts, it was found that living in a poor neighborhood and having drinkers in the social network increased relapses, while participation in Alcoholics Anonymous decreased relapses.^98^

In studies evaluating the factors affecting relapse in substance addiction, it was concluded that being single, having positive attitudes towards the substance, family conflicts, weak social support, having easy access to the substance, and having addicts in the family or close friends increased relapses.^34^ There are studies showing that positive family functioning in SUD may be effective in preventing relapses by increasing self-esteem and resilience in individuals.^99^

In a study conducted in patients with ASUD, positive family functioning in SUD was found to be associated with higher relapse rates in close relatives of patients with ASUD.^1^

In studies evaluating relapses in patients with cocaine or methamphetamine use disorder, it has been shown that social factors such as homelessness, unemployment, low family income, lack of social support, poor living and family conditions increase relapses.^47,62^

In a study conducted with 655 patients with opioid use disorder, the reasons for relapse during methadone treatment were analyzed. Long-term substance use in the past and living in mountainous regions increased relapses, while living with many family members, long treatment duration and being fully compliant with treatment decreased relapses. Gender, educational level, occupational type, monthly income of the patient, monthly income of the family, number of previous treatments, daily methadone dose and comorbidity were not found to be associated with opioid relapses among the patients.^100^

In a study of 247 patients with alcohol and substance use disorders, probation and employment were shown to reduce relapse.^46^ In a study conducted with 121 people with opioid addiction in the United States, it was shown that legal responsibility can reduce substance relapses.^101^

## Conclusion

In this study, we have tried to identify the factors that influence relapse, which is a common and important problem in ASUD. The results show that factors such as early age of onset, dysfunction of the brain reward system, poor physical health, sleep disturbance, presence of comorbid psychiatric disorders, ASUD severity, craving, smoking, low self-efficacy, negative life events and low socioeconomic status increase ASUD relapse. On the other hand, factors such as positive family functioning, strong social support, treatment motivation and regular medication seem to reduce relapse. Studies of gender, genetic and neurobiological factors have not found a consistent relationship with relapse and more research is needed. Given all this information, it is necessary to identify in detail the personal risk and protective factors that cause relapse in people with alcohol and drug addiction. New research into the factors associated with relapse will enable individualized treatment, early medical and psychological intervention, and thus better and longer-lasting treatment responses.

Compared to similar reviews in the literature, our study differs in classifying and evaluating the factors associated with relapses in AUD and SUD together and summarizing the results of recent studies. However, it is essential to note that our study has limitations, as not all relevant studies could be included, and only a summary of the literature was presented due to the comprehensive nature of the subject.
